# Transcriptional adaptations following exercise in Thoroughbred horse skeletal muscle highlights molecular mechanisms that lead to muscle hypertrophy

**DOI:** 10.1186/1471-2164-10-638

**Published:** 2009-12-30

**Authors:** Beatrice A McGivney, Suzanne S Eivers, David E MacHugh, James N MacLeod, Grace M O'Gorman, Stephen DE Park, Lisa M Katz, Emmeline W Hill

**Affiliations:** 1 Animal Genomics Laboratory, UCD School of Agriculture, Food Science and Veterinary Medicine, UCD College of Life Sciences, University College Dublin, Belfield, Dublin 4, Ireland; 2 UCD Conway Institute of Biomolecular and Biomedical Research, University College Dublin, Belfield, Dublin 4, Ireland; 3 Gluck Equine Research Center, Department of Veterinary Science, University of Kentucky, Lexington, KY 40546-0099, USA; 4 University Veterinary Hospital, UCD School of Agriculture, Food Science and Veterinary Medicine, UCD College of Life Sciences, University College Dublin, Belfield, Dublin 4, Ireland

## Abstract

**Background:**

Selection for exercise-adapted phenotypes in the Thoroughbred racehorse has provided a valuable model system to understand molecular responses to exercise in skeletal muscle. Exercise stimulates immediate early molecular responses as well as delayed responses during recovery, resulting in a return to homeostasis and enabling long term adaptation. Global mRNA expression during the immediate-response period has not previously been reported in skeletal muscle following exercise in any species. Also, global gene expression changes in equine skeletal muscle following exercise have not been reported. Therefore, to identify novel genes and key regulatory pathways responsible for exercise adaptation we have used equine-specific cDNA microarrays to examine global mRNA expression in skeletal muscle from a cohort of Thoroughbred horses (*n = *8) at three time points (before exercise, immediately post-exercise, and four hours post-exercise) following a single bout of treadmill exercise.

**Results:**

Skeletal muscle biopsies were taken from the *gluteus medius *before (T_0_), immediately after (T_1_) and four hours after (T_2_) exercise. Statistically significant differences in mRNA abundance between time points (T_0 _*vs *T_1 _and T_0 _*vs *T_2_) were determined using the empirical Bayes moderated *t*-test in the Bioconductor package Linear Models for Microarray Data (LIMMA) and the expression of a select panel of genes was validated using real time quantitative reverse transcription PCR (qRT-PCR). While only two genes had increased expression at T_1 _(*P *< 0.05), by T_2 _932 genes had increased (*P *< 0.05) and 562 genes had decreased expression (*P *< 0.05). Functional analysis of genes differentially expressed during the recovery phase (T_2_) revealed an over-representation of genes localized to the actin cytoskeleton and with functions in the MAPK signalling, focal adhesion, insulin signalling, mTOR signaling, p53 signaling and Type II diabetes mellitus pathways. At T_1_, using a less stringent statistical approach, we observed an over-representation of genes involved in the stress response, metabolism and intracellular signaling. These findings suggest that protein synthesis, mechanosensation and muscle remodeling contribute to skeletal muscle adaptation towards improved integrity and hypertrophy.

**Conclusions:**

This is the first study to characterize global mRNA expression profiles in equine skeletal muscle using an equine-specific microarray platform. Here we reveal novel genes and mechanisms that are temporally expressed following exercise providing new knowledge about the early and late molecular responses to exercise in the equine skeletal muscle transcriptome.

## Background

The Thoroughbred racehorse is an elite athlete, that for four hundred years has been selected for physiological traits enabling exceptional speed and stamina. As a highly adapted athlete the Thoroughbred is a suitable model for understanding the physiology of exercise [1]. Thoroughbreds have a very high aerobic capacity or maximal oxygen uptake (VO_2max_) [2] relative to their body mass. A bout of intense exercise requires both aerobic and anaerobic energy production and a Thoroughbred may increase its metabolic rate from basal levels by up to 60-fold under racing conditions [3]. A critical component for athletic performance is muscle and it is notable that the Thoroughbred has a high skeletal muscle mass comprising over 55% of total body mass [4].

The biological importance of skeletal muscle is reflected in its remarkable structural and functional plasticity that enables rapid alterations to phenotype following repeated bouts of exercise [5]. A single bout of acute exercise induces multiple stresses in skeletal muscle, including increased demand for ATP and mechanical stress [6,7]. The responses to these stressors can be divided into two broad categories: the return to homeostasis, and the adaptive response. The principle processes associated with homeostatic recovery are glucose sparing, elevated fat oxidation, glycogen resynthesis and free radical quenching, as well as the repairing of free radical-mediated damage and restoration of intracellular electrolyte concentrations and pH [8-12]. The adaptive response is the process whereby skeletal muscle responds to repeated exercise bouts (conditioning or training) in ways that cumulatively lead to an enhanced ability to maintain muscle homeostasis during exercise. This conditioning response involves both morphological changes, such as hypertrophy, and metabolic responses such as an increased capacity for oxidative substrate metabolism in mitochondria and a shift toward oxidizing proportionately more fats and less glucose during exercise [13,14].

Exercise studies using human subjects have demonstrated that changes in the expression of a wide range of mRNA transcripts play a major role in the adaptive response of muscle to exercise [15-18]. Furthermore, microarray studies have shown that a large number of genes are differentially expressed in skeletal muscle following exercise [19]. A single bout of exercise has been shown to increase mRNA expression particularly in genes involved in mitochondrial biogenesis and metabolism [20].

While protein changes and mRNA quantified in small panels of genes by Western blotting and real time qRT-PCR [21-24] have been investigated, global mRNA expression during the immediate-response period (< 8 minutes) has not, to our knowledge, previously been reported in skeletal muscle following exercise in any species. Also, global gene expression changes in equine skeletal muscle following exercise have not been reported. Therefore to identify novel genes and key regulatory pathways responsible for exercise adaptation we have used equine-specific cDNA microarrays to examine global mRNA expression in skeletal muscle from a cohort of Thoroughbred horses (*n = *8) at two time points (immediately, and four hours post-exercise) following a standardised incremental-step exercise test on a high-speed equine treadmill.

## Results and Discussion

### Experiment overview

Eight four-year old unconditioned Thoroughbred horses (castrated males) were exercised to maximum heart-rate or fatigue in a standardized incremental-step exercise test [25-27] on a high-speed equine treadmill. Skeletal muscle biopsy samples were collected at three time points: at rest pre-exercise (T_0_), immediately post-exercise (T_1_) and four hours post-exercise (T_2_). In a direct comparison microarray experiment, equine cDNA microarrays were hybridised with samples from T_0 _*Vs *T_1 _and from T_0 _*Vs *T_2 _for each animal.

### Exercise parameters

Following warm-up, the exercise test comprised an average of six (range 5 - 7) incremental steps achieving a mean maximum velocity of 12.4 ± 0.2 m/s and a mean distance of 4,362.9 ± 102.7 m for an average duration of 8.77 ± 0.5 min. Mean maximal heart rate was 218 ± 9 beats per minute. Mean peak post-exercise (T_1_) lactate concentrations were 13.3 ± 1.2 mmol/l and were significantly increased compared to pre-exercise values (*P *< 0.0001).

### Microarray annotation and gene ontology

Of the 9,333 ESTs on the microarray 8,519 aligned to a single location on the equine genome (EquCab 2.0), 372 aligned to more than one location and the remaining 442 failed to align to any location with high confidence. Fewer than 50% (4,631) of the ESTs matched an Ensembl gene, the majority (4,166) of which had human orthologs. The human orthologs were used to create input files for gene ontology functional analyses using the DAVID software package [28,29].

The functional representation of ESTs on the microarray relative to all genes in the *Equus caballus *Ensembl database that had human orthologs (66%) was assessed using 15 broad GO categories (developmental process, multicellular organismal process, biological regulation, metabolic process, cellular process, macromolecular complex, organelle part, organelle, cell part, cell, transporter activity, transcription regulator activity, molecular transducer activity, catalytic activity, binding). A similar distribution pattern among GO categories was observed for ESTs on the microarray when compared to all Ensembl genes (Additional file 1).

### Immediate response to exercise

#### Differential expression of genes

Immediately following exercise (T_1_) two probes were significantly (*P *< 0.05) differentially regulated. Four hours (T_2_) after exercise 1,485 probes were differentially expressed with fold changes ranging from +4.8-fold to -2.9-fold. At T_2_, 923 probes were up-regulated and 562 probes were down-regulated. At the chosen significance threshold (α = 0.05) 74 of these probes are likely to be false positives. The probes with the greatest changes in expression (> +1.5-fold) immediately post-exercise are shown in Table 1. The probes with the greatest changes in expression (> +1.5-fold or -1.5-fold) four hours post exercise are shown in Table 2 (up-regulated) and Table 3 (down-regulated). A full list of gene expression changes at T_1 _and T_2 _are available in additional files 2 and 3. The equine cDNA microarray expression data generated was deposited in the NCBI Gene Expression Omnibus (GEO) repository with experiment series accession [GEO:GSE16235].

**Table 1:** Genes ≥ +1.5-fold (up-regulated) differential expression immediately post-exercise compared to pre-exercise levels.

Gene Symbol	Gene Name	GenBank ID	Fold change	*P*	adj *P*	*A*nnotation
*HSPA1A*	Heat shock 70 kDa protein 1A	CX602571	3.11	1.50E-07	0.001	EquCab
*HSPA1A*	Heat shock 70 kDa protein 1A	CX600510	2.15	2.42E-05	0.080	EquCab
*FOS*	v-fos FBJ murine osteosarcoma viral oncogene homolog	CX597113	2.13	0.004	0.997	chr24:20,679,377-20,681,089
*PFKFB3*	6-phosphofructo-2-kinase/fructose-2,6-bisphosphatase	CX594334	1.96	4.71E-06	0.023	chr29:27,672,694-27,678,314
*FOS*	v-fos FBJ murine osteosarcoma viral oncogene homolog	CX604427	1.87	0.003	0.997	H. Sapien
*FOS*	v-fos FBJ murine osteosarcoma viral oncogene homolog	CX592361	1.59	0.039	0.997	H. Sapien
*EGR1*	early growth response 1	CX602573	1.55	0.014	0.997	H. Sapien

The gene names provided are either HUGO approved or *Equus caballus *specific.

Adj P is the P-value following adjustment for multiple testing.

Predicted gene annotations were assigned to unannotated probes of interest based on the gene located closest to the probe and homology to mammalian genes, chromosomal locations are provided for these genes.

**Table 2:** Genes ≥ +1.5-fold (up-regulated) differential expression four hours post-exercise compared to pre-exercise levels.

Gene Symbol	Gene Name	Fold change	adj *P*
*HSPA1A*	heat shock 70 kDa protein 1A	4.84	1.61E-05
*HSP90AA1*	heat shock protein 90 kDa alpha (cytosolic), class A member 1	2.20	0.002
*USP36*	ubiquitin specific peptidase 36	2.17	0.001
*VWCE*	von Willebrand factor C and EGF domains	2.07	0.001
*CCDC6*	coiled-coil domain containing 6	1.91	0.003
*HSP90AA1*	heat shock protein 90 kDa alpha (cytosolic), class A member 1	1.88	0.003
*NFIC*	nuclear factor I/C (CCAAT-binding transcription factor)	1.85	0.003
*RCSD1*	RCSD domain containing 1	1.80	0.001
*SEPT9*	septin 9	1.75	0.005
*TMEM145*	transmembrane protein 145	1.71	0.002
*BAIAP2*	BAI1-associated protein 2	1.70	0.001
*ATXN2L*	ataxin 2-like	1.70	0.003
*NUCB1*	nucleobindin 1	1.67	0.004
*HSPA8*	heat shock 70 kDa protein 8	1.67	0.005
*STRN4*	striatin, calmodulin binding protein 4	1.66	0.006
*PKM2*	pyruvate kinase, muscle	1.66	0.001
*DLX5*	distal-less homeobox 5	1.66	0.003
*CRTC2*	CREB regulated transcription coactivator 2	1.66	0.002
*C20orf112*	uncharacterised protein	1.64	0.001
*LAMP2*	lysosomal-associated membrane protein 2	1.64	0.003
*EIF1*	eukaryotic translation initiation factor 1	1.64	0.018
*PKM2*	pyruvate kinase, muscle	1.63	0.028
*PLOD1*	procollagen-lysine 1, 2-oxoglutarate 5-dioxygenase 1	1.63	0.003
*EML3*	echinoderm microtubule associated protein like 3	1.62	0.006
*C14orf43*	uncharacterised protein	1.61	0.016
*PDCD6IP*	programmed cell death 6 interacting protein	1.61	0.001
*OGFR*	opioid growth factor receptor	1.61	0.003
*EMP3*	epithelial membrane protein 3	1.61	0.006
*DRAP1*	DR1-associated protein 1 (negative cofactor 2 alpha)	1.61	0.001
*C14orf151*.	uncharacterised protein	1.59	0.004
*C20orf29*	uncharacterised protein	1.59	0.001
*TUBB*	tubulin, beta	1.58	0.001
*RNF19B*	ring finger protein 19B	1.58	0.017
*RCN3*	reticulocalbin 3, EF-hand calcium binding domain	1.58	0.004
*PRELP*	proline/arginine-rich end leucine-rich repeat protein	1.58	0.012
*PEX16*	peroxisomal biogenesis factor 16	1.58	0.003
*NR4A1*	nuclear receptor subfamily 4, group A, member 1	1.58	0.006
*FTH1*	ferritin, heavy polypeptide 1	1.58	0.004
*CRTC2*	CREB regulated transcription coactivator 2	1.58	0.004
*SUZ12*	suppressor of zeste 12 homolog (Drosophila)	1.57	0.001
*SF3B5*	splicing factor 3b, subunit 5, 10 kDa	1.57	0.003
*SLC16A3*	solute carrier family 16, member 3 (monocarboxylic acid transporter 4)	1.57	0.017
*PKM2*	pyruvate kinase, muscle	1.57	0.006
*DNAJC1*	DnaJ (Hsp40) homolog, subfamily C, member 1	1.57	0.002
*DLX5*	distal-less homeobox 5	1.57	0.004
*DCHS1*	dachsous 1 (Drosophila)	1.57	0.002
*SIPA1L1*	signal-induced proliferation-associated 1 like 1	1.56	0.014
*SNAI1*	snail homolog 1 (Drosophila)	1.55	0.006
*PRKCSH*	protein kinase C substrate 80K-H	1.55	0.007
*LCN2*	lipocalin 2	1.55	0.002
*HOXA5*	homeobox A5	1.55	0.004
*KDELR1*	KDEL (Lys-Asp-Glu-Leu) endoplasmic reticulum protein retention receptor 1	1.54	0.006
*IDH2*	isocitrate dehydrogenase 2 (NADP+), mitochondrial	1.54	0.011
*AUP1*	ancient ubiquitous protein 1	1.54	0.048
*SLC16A13*	solute carrier family 16, member 13 (monocarboxylic acid transporter 13)	1.53	0.008
*NINJ1*	ninjurin 1	1.53	0.003
*HMOX2*	heme oxygenase (decycling) 2	1.53	0.020
*HSP90AA1*	heat shock protein 90 kDa alpha (cytosolic), class A member 1	1.53	0.049
*CCDC12*	coiled-coil domain containing 12	1.53	0.011
*ARHGEF19*	Rho guanine nucleotide exchange factor (GEF) 19	1.52	0.002
*NAGPA*	N-acetylglucosamine-1-phosphodiester alpha-N-acetylglucosaminidase	1.52	0.005
*PTTG1IP*	pituitary tumor-transforming 1 interacting protein	1.51	0.014
*NR1D1*	nuclear receptor subfamily 1, group D, member 1	1.51	0.020
*CLSTN1*	calsyntenin 1	1.51	0.002
*BAIAP2*	BAI1-associated protein 2	1.51	0.005
*ATF4*	activating transcription factor 4 (tax-responsive enhancer element B67)	1.51	0.005
*C11orf24*	uncharacterised protein	1.50	0.005
*TSPAN4*	tetraspanin 4	1.50	0.008
*GMPPA*	GDP-mannose pyrophosphorylase A	1.50	0.016
*CHAD*	chondroadherin	1.50	0.005
*BCKDK*	branched chain ketoacid dehydrogenase kinase	1.50	0.014
*ACTN1*	actinin, alpha 1	1.50	0.006

Adj P is the P-value following adjustment for multiple testing.

**Table 3:** Genes ≥ -1.5-fold (down-regulated) differential expression four hours post-exercise compared to pre-exercise levels.

Gene Symbol	Gene Name	Fold change	adj *P*
*ACTR10*	actin-related protein 10 homolog (S. cerevisiae)	-1.73	0.042
*ANXA7*	annexin A7	-1.73	0.039
*CBX3*	chromobox homolog 3 (HP1 gamma homolog, Drosophila)	-1.51	0.010
*C12orf57*	chromosome 12 open reading frame 57	-1.52	0.008
*C17orf37*	chromosome 17 open reading frame 37	-1.59	0.011
*COPB2*	coatomer protein complex, subunit beta 2 (beta prime)	-1.55	0.009
*CFH*	complement factor H	-1.63	0.035
*CWF19L2*	CWF19-like 2, cell cycle control (S. pombe)	-2.87	2.67E-03
*CWF19L2*	CWF19-like 2, cell cycle control (S. pombe)	-2.14	0.003
*CYCS*	cytochrome c, somatic	-1.52	0.012
*FBXW5*	F-box and WD repeat domain containing 5	-1.57	0.007
*GALM*	galactose mutarotase (aldose 1-epimerase)	-1.62	0.023
*GLB1*	galactosidase, beta 1	-1.54	0.006
*GNL3*	guanine nucleotide binding protein-like 3 (nucleolar)	-1.54	0.019
*HBS1L*	HBS1-like (S. cerevisiae)	-1.70	0.042
*HBS1L*	HBS1-like (S. cerevisiae)	-1.62	0.047
*KLHL2*	kelch-like 2, Mayven (Drosophila)	-1.66	0.040
*LRRC8D*	leucine rich repeat containing 8 family, member D	-1.67	0.039
*ME1*	malic enzyme 1, NADP(+)-dependent, cytosolic	-1.59	0.003
*MUT*	methylmalonyl Coenzyme A mutase	-1.59	0.014
*MIPEP*	mitochondrial intermediate peptidase	-1.69	0.012
*MRPL39*	mitochondrial ribosomal protein L39	-1.52	0.013
*NDUFA12*	NADH dehydrogenase (ubiquinone) 1 alpha subcomplex, 12	-1.50	0.020
*NDN*	necdin homolog (mouse)	-2.01	0.004
*NEDD1*	neural precursor cell expressed, developmentally down-regulated 1	-1.52	0.022
*PCOLCE2*	procollagen C-endopeptidase enhancer 2	-2.10	0.004
*PCOLCE2*	procollagen C-endopeptidase enhancer 2	-1.62	0.010
*PCOLCE2*	procollagen C-endopeptidase enhancer 2	-1.51	0.017
*QKI*	quaking homolog, KH domain RNA binding (mouse)	-1.54	0.020
*RTN4*	reticulon 4	-1.59	0.039
*RPL22*	ribosomal protein L22	-1.61	0.048
*ROBO1*	roundabout, axon guidance receptor, homolog 1 (Drosophila)	-1.83	0.007
*SIAH2*	seven in absentia homolog 2 (Drosophila)	-1.55	0.023
*TXNDC17*	thioredoxin domain containing 17	-2.15	0.005
*TRAM1*	translocation associated membrane protein 1	-1.87	0.008
*UXS1*	UDP-glucuronate decarboxylase 1	-2.21	0.016
*C13orf8*	uncharactherised protein	-1.51	0.020
*VPS33A*	vacuolar protein sorting 33 homolog A (S. cerevisiae)	-1.70	0.048

Adj P is the P-value following adjustment for multiple testing.

Among the probes with the greatest expression changes (> +1.5-fold) at T_1_were seven probes representing four genes: three probes representing *FOS *(v-fos FBJ murine osteosarcoma viral oncogene homolog gene; mean +1.9-fold, unadjusted *P *= 0.004, 0.003, 0.039); two probes representing *HSPA1A *(heat shock 70 kDa protein 1A gene; mean +2.7-fold, unadjusted *P *= 1.50E-07, 2.42E-05); one probe located ~ 2kb upstream of *PFKFB3 *(6-phosphofructo-2-kinase/fructose-2,6-biphosphatase 3 gene; +2.0-fold, unadjusted *P = *4.71E-06) and one probe representing *EGR1 *(early growth response 1 gene; +1.6-fold, unadjusted *P = *0.014).

The gene expression changes observed for the *FOS *and *HSPA1A *genes are consistent with previous mammalian studies that have shown increased expression of these genes in response to exercise [24,30]. *HSPA1A, FOS *and *EGR1 *are members of the immediate-early response (IER) gene family. These genes are early regulators of cell growth and differentiation signals, and are induced in response to a wide variety of stress stimuli [31]. The heat shock protein Hsp70, encoded by the *HSPA1A *gene, is known to protect skeletal muscle cells against the path physiological effects of oxidative stress. In transgenic mouse models this cytoprotection is brought about both through improvement in muscle function and decreased apoptosis [32-34]. It has been suggested that the cytoprotective effects of the Hsp70 protein are related to an ability to assist with the refolding of denatured or partially degraded proteins [35]. Hsp70 can also interact with proteins involved in the regulation of cellular redox balance and Ca^2+ ^homeostasis, and thus reduce oxidative stress and Ca^2+ ^overload in response to physiological stress [36]. In addition Hsp70 protects against muscular degeneration and atrophy [37] through inhibition of caspase activation [38] and protein catabolism [37] and Hsp70 protein levels have been shown to correlate with muscular regeneration following injury [39]. Together these facts highlight the key role of Hsp70 in muscle protection following stress and as a modulator of muscular regeneration. The *HSPA1A *gene displayed a further increase in transcript expression at T_2 _(+4.8-fold, *P *< 0.001), whereas the expression of *FOS, EGR1 *and *PFKFB3 *had returned to resting levels. This suggests that while *FOS*, *EGR1 *and *PFKFB3 *responses may be immediate and transient, the *HSPA1A *response likely contributes to long term adaptation.

The probe upstream of the *PFKFB3 *gene shares strong homology to mammalian homologues of the gene thus it is likely that it represents expression of this gene product. The product of the *PFKFB3 *gene is involved in various aspects of energy sensing and metabolism, but has not previously been shown to be increased due to exercise. However, studies have shown increased expression of *PFKFB3 *in response to glucose deprivation [40] and hypoxia [41], both stimuli associated with exercise. The PFKFB3 protein is a powerful activator of glycolysis [42]. Surprisingly, in a panel of genes encoding glycolytic enzymes and other anaerobic metabolites, differential mRNA expression was not observed in this experimental cohort despite significant increases in plasma lactate concentrations [43]. Similar observations of a lack of transcriptional activation of glycolytic genes have been made in human exercise studies [44]. PFKFB3 is also involved in glucose-induced insulin secretion in pancreatic β cells [45] and a SNP in the 3' untranslated region of the *PFKFB3 *gene is associated with obesity in humans [46]. The *PFKFB3 *gene promoter contains hypoxic response elements necessary for transactivation by hypoxia-inducible factor-1 alpha (HIF-1α) in response to hypoxia [47]. This is relevant considering the observed increase in HIF-1α protein in this cohort of horses immediately after exercise [43].

There was some overlap among probes differentially expressed at T_2 _and those tending towards differential expression at T_1_. Among the 434 probes tending towards differential expression (unadjusted *P *< 0.05) at T_1 _154 were also among those at T_2_, which is more than twice as many expected by chance. Over 96% of the genes had both the same direction of regulation at both time-points and a greater magnitude of change at T_2_. Two genes had a greater magnitude of change at T_1 _and a different directionality was observed for four genes. The genes with the highest observed fold changes at both T_1 _and T_2 _included *HSPA1A *(heat shock 70 kDa protein 1A gene, T_1_: +2.6-fold (mean of two probes), unadjusted *P *= 1.22E-05; T_2_: +4.8-fold, *P *= 1.61E-05); *CRTC2 *(CREB regulated transcription coactivator 2 gene, T_1_: +1.3-fold, adjusted *P *= 0.001; T_2_: +1.7-fold, *P *= 0.003); and *SLC16A3 *(solute carrier family 16, member 13 gene, T_1_: +1.2-fold, adjusted *P *= 0.03; T_2_: +1.6-fold, *P *= 0.012).

The CRTC2 protein is a potent activator of PGC-1α (peroxisome proliferater-activated receptor gamma coactivator 1 alpha), the master regulator of mitochondrial biogenesis [48] and is also involved in the modulation of gluconeogenesis [49]. The SLC16A3 protein is found in greater abundance in fast twitch rather than slow twitch muscle [50] and plays a direct role in lactate efflux out of skeletal muscle. Thoroughbred horses have a strikingly high proportion of fast to slow twitch muscle fibres [51], which was also observed in this cohort of horses [43]. Increased mRNA levels of *SLC16A3 *were observed in "race fit" compared to moderately conditioned Standardbred horses [52]. SLC16A3 also plays a role in the transport of the performance enhancing drug gamma-hydroxybutyric acid (GHB) [53]. GHB is an endogenous metabolite but can also be administered orally as a performance-enhancing drug; therefore it is reasonable to hypothesize that endogenous GHB metabolism is associated with natural athletic ability. This hypothesis is supported by the observation that the alcohol dehydrogenase iron-containing protein 1 gene (*ADHFE1*), which is involved in GHB catabolism [54] is located in a genomic region that has been a target for positive selection during four hundred years of Thoroughbred evolution [55].

Overall, these data suggest that, in addition to a rapid and dramatic induction of a small number of stress response genes immediately after exercise, there are also more subtle early changes in gene expression that are difficult to detect but are functionally relevant. It is possible that many of the genes differentially expressed at T_2 _were also differentially expressed at T_1_, but show more gradual changes in gene expression and were not detectable at that time point.

#### Overrepresentation of functional ontologies among differentially expressed genes

The relatively small number of probes (*n *= 434, unadjusted *P *< 0.05) tending towards significant differential expression immediately after exercise suggested that deriving meaningful functional information may be problematic given an expected false discovery rate of approximately 400 probes in this experiment. Therefore the FatiScan gene enrichment test, which incorporates all transcriptional data rather than limiting to only significantly differentially expressed probes was used to analyse the transcriptional profile immediately after exercise [56]. Genes were ranked by differential expression and functional blocks that were significantly up-regulated and down-regulated immediately after exercise were identified (Table 4). Overrepresented GO functional groups associated with up-regulated genes included response to stress, RNA metabolism and developmental processes. The overrepresentation of genes involved in the stress response suggests that exercise-induced muscle repair may be a particular requirement for the maintenance of structural integrity in Thoroughbred skeletal muscle following disruption of muscle fibres. This may be understood in the light of very high aerobic and anaerobic capacities in Thoroughbreds, which enable high intensity exercise even in the unconditioned state. The principal GO functional groups associated with down-regulated genes were those involving the ribosome, oxidative phosphorylation and proton-transporting ATP synthase complex. The strong overrepresentation of down-regulated ribosomal genes suggests an inhibition of protein synthesis. Previous studies have reported a reduced rate of protein synthesis [57,58] and observed the disaggregation of polysomes to ribosomes immediately post exercise [59]. The down-regulation of genes associated with oxidative phosphorylation may represent a shift form aerobic towards anaerobic respiration.

**Table 4:** Significantly up-regulated and down-regulated GO categories immediately post-exercise compared to pre-exercise levels.

GO ID Up-regulated	GO Term	No. Genes	*P*
GO:0006950	response to stress	3	0.001
GO:0007275	multicellular organismal development	6	0.001
GO:0048731	system development	5	0.002
GO:0048856	anatomical structure development	5	0.003
GO:0009986	cell surface	12	0.005
GO:0007242	intracellular signaling cascade	195	0.009
GO:0009897	external side of plasma membrane	5	0.026
GO:0005794	Golgi apparatus	76	0.034
GO:0044459	plasma membrane part	53	0.039
GO:0016070	RNA metabolic process	4	0.040
**Down-regulated**			
GO:0003735	structural constituent of ribosome	21	0.000
GO:0005840	ribosome	26	0.000
GO:0044445	cytosolic part	33	0.001
GO:0043228	non-membrane-bound organelle	187	0.001
GO:0015934	large ribosomal subunit	15	0.003
GO:0043232	intracellular non-membrane-bound organelle	187	0.004
GO:0005829	cytosol	130	0.007
GO:0006119	oxidative phosphorylation	28	0.039
GO:0045259	proton-transporting ATP synthase complex	8	0.043

The FatiScan gene enrichment test was used to analyse the data

The majority of exercise studies investigating the immediate response to exercise have focussed on post-transcriptional or post-translational modifications or have used real time qRT-PCR to investigate a limited number of genes [21-23,60]. Because of the lack of literature documenting the immediate transcriptional response to exercise in skeletal muscle it is not clear whether the responses detected in this study are specific to horses, or indeed Thoroughbred horses.

### Delayed response to exercise

#### Differentially expressed genes

A significantly larger number of genes were differentially expressed four hours following exercise. Sixteen genes had very significant (*P *< 0.01) increases in expression with magnitudes > +1.8-fold and 104 had had significant (*P *< 0.05) expression differences > +1.5-fold. Among the 16 most differentially expressed genes at T_2 _were *HSPA1A *(heat shock 70 kDa protein 1A gene, +4.8-fold, *P *< 0.001); *TPM4 *(tropomyosin 4 gene; +1.9-fold, *P *= 0.008), *HSP90AA1 *(heat shock protein 90 kDa alpha (cytosolic), class A member 1 gene; +2.2-fold, *P *= 0.002) and *USP36 *(ubiquitin specific peptidase 36 gene; +2.16-fold, *P *= 0.001). Other notable genes present among those differentially upregulated (> +1.5-fold) were *HSPA8 *(heat shock 70 kDa protein 8 gene; +1.9-fold, *P *= 0.003); *CRTC2 *(CREB-regulated transcription coactivator 2 gene; +1.7-fold, *P *= 0.002) and *LAMP2 *(lysosome-associated membrane glycoprotein 2 precursor gene; +1.6-fold, *P *= 0.028.

Notably genes encoding three heat shock proteins (*HSPA1A*, *HSP90AA1 *and *HSPA8*) were among the most highly differentially regulated transcripts. The Hsp70 (*HSPA1A*) and Hsp90 (*HSP90AA1*) proteins have been shown to be associated with the transport of TOM (Translocases of the outer membrane) complex proteins to the mitochondrial surface [61,62] in response to contractile activity. These proteins in turn are responsible for the import of the hundreds of nuclear encoded proteins that function in the mitochondria [63,64]. It clearly follows that the reliance on nuclear encoded proteins for mitochondrial function is subject to the efficiency of protein translocation to the mitochondria While numerous studies have reported Hsp70 and Hsp90 induction in skeletal muscle in response to exercise [65,66], to our knowledge, no study has reported an induction of constitutively expressed HSPA8 protein. On the other hand, HSPA8 has been reported to be induced in rat cardiac muscle following hypoxic exposure [67] and may provide a protective effect following oxidative stress [68]. LAMP-2 is a lysosomal receptor involved in the elimination of misfolded proteins. It has been demonstrated that protesomal inhibition results in an accumulation of Hsp70, LAMP-2 and ubiquitin aggregates [69]. Similarly, the TPM4 protein plays a major role in Ca^2+^-regulated skeletal muscle contraction and is upregulated in muscle undergoing regeneration and focal repair [70]. Presumably the up-regulation of the heat shock genes, *TPM4*, *LAMP2 *and *USP36 *reflects activity in the reparation or degradation of damaged and misfolded proteins [69].

Fourteen probes representing 12 genes had very significant (*P *< 0.01) decreases in expression at T_2 _with magnitudes greater than -1.8-fold. One hundred and twenty-six genes had significant (*P *< 0.05) expression differences greater than -1.5-fold. The most differentially expressed genes were *CWF19L2 *(CWF19-like protein gene;represented by two probes, mean -2.5-fold, *P *= 0.003); *UXS1 *(UDP-glucuronic acid decarboxylase 1 gene; -2.2-fold, *P *= 0.016); *TXNL5 *(thioredoxin domain-containing protein 17 gene; -2.2-fold, *P *= 0.005); *PCOLCE2*, (procollagen C-endopeptidase enhancer 2 precursor gene; represented by two probes, mean -1.9-fold, *P *= 0.004, *P *= 0.01); *NDN *(necdin gene; -2.0-fold, *P *= 0.004); *TRAM1 *(translocation-associated membrane protein 1 gene; -1.9-fold,*P *= 0.008); and *ROBO1 *(roundabout homolog 1 precursor gene; -1.8-fold, *P *= 0.007). Six probes also had decreased expression at T_1 _(unadjusted *P *< 0.05) representing *GLB1 *(T_1_: -1.2-fold; T_2_: -1.5-fold), *SETD7 *(T_1_: -1.1-fold; T_2_: -1.5-fold) and four unannotated probes.

#### Overrepresentation of functional ontologies among differentially expressed genes

At T_2 _there was an observed overrepresentation of genes that localised to the actin cytoskeleton, actin filament bundle and cortical actin cytoskeleton (Table 5). The overrepresentation of genes associated with the actin cytoskeleton may be indicative of responses to contraction and mechanical stimuli and may be associated with muscle remodelling via sarcomerogenesis. This is consistent with an observed overrepresentation of genes in the focal adhesion pathway. Actin remodelling has also been shown to be responsible for an increase in GLUT4 translocation in skeletal muscle [71]. An overrepresentation of actin-related gene ontologies following exhaustive exercise has not previously been reported. On the other hand, the observed overrepresentations of genes with intramolecular oxidoreductase activity, unfolded protein binding and heat shock protein binding molecular functions are consistent with human exercise studies that predict replenishment of intramuscular energy stores and a stress response during recovery from intense exercise because of ROS production, inflammation and intramuscular microtears [8,19].

**Table 5:** GO categories and KEGG pathways overrepresented among differentially expressed genes (*P *≤ 0.05) four hours post-exercise compared to pre-exercise levels.

GO ID Molecular Function	GO Term	No. Genes	*P*	Fold Enrichment
GO:0005515	protein binding	352	0.004	1.09
GO:0016853	isomerase activity	19	0.007	1.85
GO:0005200	structural constituent of cytoskeleton	7	0.026	2.72
GO:0003700	transcription factor activity	39	0.031	1.36
GO:0016860	intramolecular oxidoreductase activity	8	0.037	2.33
GO:0031072	heat shock protein binding	9	0.078	1.91
GO:0051082	unfolded protein binding	16	0.096	1.49
**Cellular Compartment**				
GO:0044428	nuclear part	83	0.009	1.27
GO:0044446	intracellular organelle part	194	0.015	1.13
GO:0044422	organelle part	195	0.016	1.13
GO:0031974	membrane-enclosed lumen	75	0.018	1.26
GO:0043233	organelle lumen	75	0.018	1.26
GO:0005667	transcription factor complex	19	0.024	1.67
GO:0015629	actin cytoskeleton	15	0.029	1.78
GO:0044451	nucleoplasm part	37	0.032	1.37
GO:0005886	plasma membrane	79	0.042	1.20
GO:0016020	membrane	222	0.049	1.09
GO:0005654	nucleoplasm	40	0.061	1.29
GO:0031981	nuclear lumen	51	0.064	1.24
GO:0001725	stress fiber	4	0.066	3.79
GO:0032432	actin filament bundle	4	0.066	3.79
GO:0030864	cortical actin cytoskeleton	4	0.066	3.79
GO:0030863	cortical cytoskeleton	5	0.066	2.96
GO:0030054	cell junction	15	0.074	1.58
GO:0044433	cytoplasmic vesicle part	10	0.077	1.82
GO:0030659	cytoplasmic vesicle membrane	10	0.077	1.82
GO:0044448	cell cortex part	6	0.087	2.37
GO:0031410	cytoplasmic vesicle	28	0.090	1.33
GO:0012506	vesicle membrane	10	0.095	1.75
GO:0016023	cytoplasmic membrane-bound vesicle	24	0.098	1.35
GO:0005770	late endosome	7	0.100	2.07
**Biological Process**				
GO:0051640	organelle localization	5	0.021	3.92
GO:0006457	protein folding	29	0.029	1.45
GO:0051650	establishment of vesicle localization	4	0.032	4.70
GO:0051648	vesicle localization	4	0.032	4.70
GO:0006903	vesicle targeting	4	0.032	4.70
GO:0050790	regulation of catalytic activity	26	0.044	1.44
GO:0051726	regulation of cell cycle	34	0.045	1.36
GO:0000074	regulation of progression through cell cycle	34	0.045	1.36
GO:0048523	negative regulation of cellular process	64	0.050	1.22
GO:0016568	chromatin modification	19	0.058	1.51
GO:0051656	establishment of organelle localization	4	0.067	3.76
GO:0065009	regulation of a molecular function	29	0.076	1.34
GO:0048519	negative regulation of biological process	65	0.080	1.19
GO:0007049	cell cycle	49	0.080	1.23
GO:0031324	negative regulation of cellular metabolic process	25	0.082	1.37
**KEGG ID**				
hsa04010	MAPK signaling pathway	13	0.060	1.69
hsa04510	Focal adhesion	17	0.099	1.45
hsa04115	p53 signaling pathway	5	0.163	2.24
hsa00590	Arachidonic acid metabolism	3	0.182	3.70
hsa04910	Insulin signaling pathway	9	0.183	1.59
hsa05211	Renal cell carcinoma	7	0.198	1.73
hsa04670	Leukocyte transendothelial migration	7	0.233	1.64
hsa04150	mTOR signaling pathway	4	0.265	2.19
hsa00720	Reductive carboxylate cycle (CO2 fixation)	3	0.265	2.96
hsa04930	Type II diabetes mellitus	3	0.265	2.96

The FatiScan gene enrichment test was used to analyse the data

Because of the larger number of genes with assigned biological processes (497) the returned GO classes had more general higher level functions (*e.g*. protein folding, regulation of catalytic activity and regulation of the cell cycle) providing little insight into the underlying adaptive mechanisms. Therefore, we searched for overrepresented KEGG pathways among the significantly differentially regulated genes at T_2. _These included the well established exercise response pathways, insulin signalling [68], Type II diabetes mellitus [72], mTOR signalling [73] and MAPK signaling [74-76] as well as focal adhesion and p53 signaling pathway. A list of genes differentially expressed in these pathways is provided in additional file 4. The overrepresented KEGG pathways are associated with different but overlapping aspects of exercise stimuli and support the hypothesis that the genes governing these cellular pathways have been targets for selection for exercise adaptation in Thoroughbreds [55].

The less well described focal adhesion and p53 signaling pathways are of particular note for their roles in muscle hypertrophy and metabolic improvements. Muscle stretch gives rise to the generation of focal adhesion complexes through the induction of actin polymerisation at focal adhesions and an increase in focal adhesion complex associated proteins has been found in hypertrophic muscle [77,78]. For instance, the mechanosensitive extra cellular matrix protein tenascin-C has been identified as a critical regulator of gene expression relating to repair and growth in muscle following damaging exercise [79]. Furthermore, focal adhesion kinase (FAK) has been shown to be an upstream regulator of the control of muscle mass via p70S6K [80] which may signal mTOR [81] independent of Akt. The central role of FAK in muscle growth and differentiation has been recently been demonstrated. Over-expression of FAK led to a shift towards slow twitch muscle generation and an up-regulation of genes involved in mitochondrial metabolism and contraction [82]. Therefore, an overrepresentation of focal adhesion molecules indicates the importance of mechanical force altering skeletal muscle gene regulation towards muscle growth and remodelling [83]. Moreover, we have previously determined that focal adhesion molecules may represent targets for recent artificial selection in the Thoroughbred and therefore may be critical to the development of the muscle strength phenotype for which Thoroughbreds are renowned [55]. The p53 protein is best known for its role in apoptosis, however, recent studies have suggested that the p53 signaling pathway may play a role in regulation of aerobic metabolism with significant reductions in COX4 activity in KO mice [84,85]. Importantly, p53 may regulate the expression of PGC-1α [86].

Other KEGG pathways that were overrepresented were: arachidonic acid metabolism, involved in the modulation of function of voltage gated ion channels, primarily in neurons and muscle cells [87]; leukocyte transendothelial migration, associated with the inflammatory response and largely coordinated by chemokines [88,89]; reductive carboxylate cycle (CO_2 _fixation), a metabolic pathway; and the renal cell carcinoma signalling pathway, which involves increased cell proliferation, energy demand and O_2 _usage and is stimulated by hypoxia and HIF-1α [90,91].

### Validation of a panel of genes by real time qRT-PCR

**Figure 1:**
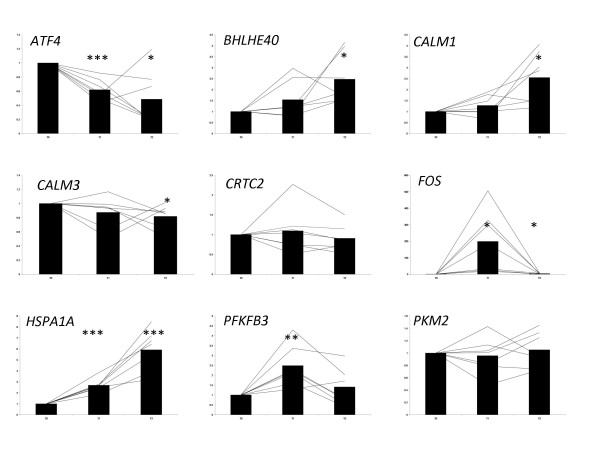
**Real time qRT-PCR results for genes used to validate microarray data**. The standard 2^-ΔΔCT ^method was used to determine mean fold changes in gene expression [116]. All Ct values were normalised using the *NSUN6 *gene. The Student's t-test was used to identify significant differences in mRNA abundance between time-points. Each point on the graph represents the relative fold change in gene expression compared to pre-exercise levels. * signifies a P-value of <0.05 **, signifies a P-value of <0.01, *** signifies a P-value of <0.001

Nine genes that were found to be differentially expressed in the microarray experiment were selected for validation by real time qRT-PCR. These genes were chosen based on their involvement in muscle contraction or the response to hypoxia. Two probes (Genbank IDs: CX594334 and CX598227) that showed differential expression, but were not found within an annotated gene were also included for validation. CX594334 lies ~ 2 kb upstream of *PFKFB3 *and was upregulated immediately post-exercise. CX598227 lies ~ 1 kb downstream of calmodulin 1 (*Calm1*) and was upregulated four hours post exercise. The average gene expression of seven of the nine probes studied reached significance (*P *< 0.05) and six [(basic helix-loop-helix family, member e40 (*BHLHE40*), calmodulin 3*(CALM3), HSPA1A, FOS*, CX594334 and *CALM1*] were concordant with the microarray data (Table 6, Figure 1). A point of major concern in microarray studies is the presence of false positives within a gene list. Although the use of qRT-PCR is essential to validate the overall dataset it is not feasible to interpret the experimental findings by evaluating each gene individually. As genes function in co-operation within complex networks we report principally the expression patterns of functionally related groups of genes.

**Table 6:** Real time qRT-PCR results for genes used to validate microarray data

Gene symbol	GenBank ID	Microarray		qRT-PCR	
		**FC T**_1_	*P*	**FC T**_1_	*P*
*HSPA1A*	CX602571	3.11	0.001	2.68	2.87E-04
PFKFB3	CX594334	1.84	0.023	2.30	0.010
*FOS*	CX604427	2.13	0.997	198.90	0.031
		**FC T**_2_	***P***	**FC T**_2_	***P***
*ATF4*	CX603997	1.50	0.005	-2.04	0.014
*BHLHE40*	CX599005	1.44	0.008	2.25	0.014
*CALM3*	CX600504	-1.40	0.045	-1.22	0.031
*CRTC2*	CX604423	1.58	0.004	-1.12	0.483
*Calm1*	CX598227	1.49	0.004	2.36	0.010
*HSPA1A*	CX602571	4.84	1.6E-05	5.91	4.08E-04
*PKM2*	CX594899	1.63	0.028	1.05	0.648

FC T_1 _represents the fold change in gene expression immediately post-exercise.

FC T_2 _represents the fold change in gene expression four hours exercise post-exercise.

There were however, some interesting findings among the validated genes. For instance, the expression of *FOS *showed a high inter-sample variance in gene expression change estimates (+18.2-fold to +506.0-fold increase). The mean expression of the *FOS *gene mRNA transcript increased +198-fold. The biological significance of the high gene expression variance for this gene is not clear at present but warrants further investigation.

Transcripts for the *HSPA1A *gene and the probe CX594334 (which may represent the *PFKFB3 *gene) were significantly increased at T_1 _(2.3-fold and 2.7-fold fold respectively). While both genes have quite different physiological roles [42,92] both contain hypoxic response elements (HRE) and have been shown to be transcriptionally activated by the HIF-1α protein under hypoxic conditions [93].

During the recovery period, four hours post exercise, *HSPA1A *mRNA levels remained elevated (+5.9-fold) while CX594334 transcript levels returned to baseline (Figure 1). The *BHLHE40 *gene, which increased in expression +2.3-fold is a transcription factor involved in the hypoxic response, contains a HRE and is inducible in hypoxic conditions through interaction with HIF-1α [94,95]. *CALM3 *and *CX598227 *which lies ~ 1 kb downstream of *CALM1 *showed directionally different changes in gene expression. *CALM3 *was downregulated -0.82-fold while *CX598227 *was upregulated +2.4-fold. This is of particular interest as little is known regarding the differential regulation of the individual genes within the Calmodulin gene family. Calmodulin is a calcium binding protein which acts as a calcium sensor [96] and plays an important role in mediating many cellular processes including muscle contraction [97,98].

## Conclusion

The Thoroughbred horse provides a singular model system to understand exercise adaptations. For the first time following exhaustive exercise we have identified a large number of genes with functions in mechanosensation, muscle hypertrophy, repair and remodelling. The induction of the large numbers of genes with such functions may be explained by the extraordinary innate aerobic and anaerobic capacity of Thoroughbreds enabling high intensity exercise even in an unconditioned state leading to proportionally greater stresses on peripheral systems than in other species. Importantly it is unlikely this knowledge could be readily gained from human studies as the sustained "all out" effort required to elicit such molecular responses is difficult to attain from untrained/sedentary human subjects, but is naturally achieved by Thoroughbreds.

The standard exercise test employed in equine exercise physiology studies requires both endurance and strength, a combination that is not easily reconstructed in other exercise models. The result is that immediately after exercise ribosomal genes are down-regulated indicating decreased protein synthesis, a signature of endurance exercise. However, established responses associated with resistance exercise such as muscle repair and hypertrophy are observed four hours after exercise. Although the inhibition of protein synthesis and muscle hypertrophy are established responses to endurance and resistance exercise respectively here we detect both responses at a global transcriptional level from a single exercise bout combining both endurance and resistance stimuli.

This study has provided a snapshot of the transcriptional response to exercise in skeletal muscle from a highly adapted system. Genes that were differentially expressed immediately after exercise are likely to be directly involved in metabolism and the stress response. Four hours following exercise a more general transcriptional response associated with recovery and adaptation was observed, in particular highlighting the roles of genes in metabolism and muscle hypertrophy. Further studies are needed to clearly distinguish between the mechanisms associated with the recovery from exercise and return to homeostasis and those that are involved in the long term adaptive response to recurring bouts of exercise conditioning.

## Methods

All animal procedures were approved by the University College Dublin, Animal Research Ethics Committee. In addition, a licence was granted from the Department of Health and Children (Ireland) and owners' consent was obtained for all horses.

### Subjects

Eight four-year old unconditioned Thoroughbred horses (castrated males), raised at the same farm for the previous 12 months and destined for National Hunt racing with the same trainer comprised the study cohort. The horses had a mean height of 165.25 cm ((± 1.44) and a mean pre-exercise weight of 565.75 kg (± 13.71). All horses participated in a standardized incremental-step exercise test [25-27] on a high-speed equine treadmill (Sato, Sato AB, Knivsta, Sweden). Before the exercise test, all horses were judged to be clinically healthy based on a veterinary examination that included a lameness assessment, resting upper airway endoscopy and basic bloodwork (complete blood count and serum biochemistry). Prior to entering the study, all of the horses had been raised together and had been kept in a grass field and fed 1.8 kg of 14% Race horses cubes (Gain horse feeds, Clonroche, Co. Wexford, Ireland) three times a day. During the study week the horses were housed in a stable and provided *ab libitum *access to water and fed grass hay and 2 kg of 10% Cool-n-Cooked Horse and Pony Mix (Connolly's Red Mills, Bagnelstown, Co. Carlow, Ireland) twice daily. Horses were fed approximately 3 hours and 55 minutes (235 ± 0.11 minutes) prior to the exercise test. All exercise tests were performed between 1000 - 1130 am.

### Standardised exercise test

The treadmill was housed in an insulated room with temperature and humidity monitors. Prior to the exercise test, all horses were acclimatized to stand quietly and to comfortably transition gaits on the treadmill. The treadmill was set to a 6° incline for all of the exercise tests. The warm-up period consisted of 2 minutes at 2 m/s, followed by 2 minutes at 4 m/s and then 2 minutes at 6 m/s. This was then followed by an increase in treadmill velocity to 9 m/s for 60 seconds, and then a 1 m/s increase in treadmill velocity every 60 seconds until the animal was no longer able to maintain its position on the treadmill at that speed or until the heart rate reached a plateau (HR_max_). Following completion of the test, the horses were quickly brought back to a walk, taken off the treadmill and washed down with cold water.

#### Instrumentation

Any instrumentation was performed 30 minutes to 1 hour prior to the exercise test. Heart rate (HR) was measured continuously before, during and after exercise by telemetry (Polar Equine S810i heart rate monitor system, Polar Electro Ltd, Warwick United Kingdom). Venous blood samples were collected immediately after exercise, 5 minutes after exercise and 4 hours post-exercise. Blood samples were collected and placed into fluoride oxalate tubes for the determination of plasma lactate concentrations. All tubes were stored on ice with the plasma separated within 1 hour of collection and analyzed within 30 minutes using the YSI 2300 STAT Plus™ lactate analyzer (YSI UK Ltd, Hampshire, UK).

### Muscle biopsy sampling

Percutaneous needle muscle biopsies [99] were obtained from the dorsal compartment of the *gluteus medius *muscle according to Dingboom and colleagues [100] using a 6 mm diameter, modified Bergstrom biopsy needle (Jørgen KRUUSE, Veterinary Supplies). Biopsies were taken approximately 15 cm caudodorsal (one-third of the distance) to the *tuber coxae *on an imaginary line drawn from the *tuber coxae *to the head of the tail. The biopsies were obtained at a depth of 80 mm. Each biopsy site was shaved, scrubbed with an antiseptic and desensitized by a local anaesthetic. The biopsy samples were washed with sterile PBS (BD Biosciences, San Jose, CA) and preserved in RNA*later *(Ambion, UK) for 24 hours at 4°C and then stored at -20°C.

Muscle biopsy samples were collected at three time points: at rest pre-exercise (T_0_), immediately post-exercise (T_1_) and four hours post-exercise (T_2_). Pre-exercise biopsies were collected within 93 minutes (range 68 - 93 mins) before the commencement of exercise and between 155 and 170 minutes post feeding. The immediately post-exercise (T_1_) biopsies were collected within seven minutes 30 seconds (range 5 mins 45 sec - 7 mins 30 sec) following cessation of exercise and four hour post-exercise (T_2_) biopsies were collected within 262 minutes (range 242 - 262 mins) following cessation of exercise.

### RNA isolation and purification

Approximately 100 mg of each muscle biopsy sample was removed from RNA*later *and homogenized in 3 ml TRIzol using a Kinematica Polytron Homogeniser PT 1200 C Drive unit, 230 V (AGB, Dublin, Ireland) and the aqueous and organic phases were separated using 200 μl of chloroform. Total RNA was precipitated using isopropyl alcohol (0.6 times the volume of the aqueous phase). The remaining pellet was washed once in 75% ethanol, and redissolved in 35 μl of nuclease-free water (Promega UK Ltd, Southampton, UK). Each sample was purified using the RNeasy ^® ^Mini kit (Qiagen Ltd, Crawley, UK) and DNase treated with RNase free DNase (Qiagen Ltd, Crawley, UK). To elute the total RNA, 35 μl of nuclease-free water were applied to the silica-gel membrane of the column to elute the total RNA, which was stored at -80°C. RNA was quantified using a NanoDrop^® ^ND1000 spectrophotometer V 3.5.2 (NanoDrop Technologies, Wilmington, DE) and RNA quality was subsequently assessed using the 18S/28S ratio and RNA integrity number (RIN) on an Agilent Bioanalyser with the RNA 6000 Nano LabChip kit (Agilent Technologies Ireland Ltd, Dublin, Ireland) according to manufacturer's instructions. The RNA isolated from these samples had an average RNA integrity number (RIN) of 8.43 ± 0.08 (range 8.0 - 9.0).

### Microarray description and annotation

Microarray slides were printed with clones selected from a cDNA library generated using mRNA purified from the articular cartilage of a 15-month old male Thoroughbred horse [101]. Probe sets on the microarray slides were prepared and printed as previously described [102,103].

The cDNA sequences for all annotated genes on the *Equus caballus *Version 2.0. (EquCab2.0) genome sequence http://www.broad.mit.edu/mammals/horse/ were downloaded from Ensembl http://www.ensembl.org, release 50. The expressed sequence tag (EST) sequences of all the probes on the array were masked to remove repeats using RepeatMasker [104] and blast searched against the cDNAs. BLAST hits were filtered to retain only hits with e values ≤ 10^-10^, ≥ 50 bp long, above 95% match-target identity, and where the best hit e value was ≥ 10^10 ^better than the next best. The EST sequences were cross-matched to horse Entrez gene IDs and to human Ensembl and Entrez gene IDs via accessions. EST matches to multiple horse or human genes were excluded. Because fewer than 50% of ESTs matched an Ensembl gene predicted gene annotations were assigned to unannotated probes of interest based on the gene located closest to the probe and homology to mammalian genes. Following a BLAST of RefSeq and protein databases search hits with an e value of < 1^-10 ^and hits on non-mammalian species were eliminated. If the predicted gene annotations based on location and homology did not match, the probe was not assigned a predicted annotation.

### Microarray hybridisation and experimental design

Total RNA was amplified using a MessageAmpTM amplified RNA (aRNA) linear amplification kit (Ambion). 2 μg of aRNA was reverse transcribed and directly labelled with Fluor647 or Fluor555 using the SuperScript™ Plus Direct cDNA Labeling System (Bio-sciences, Dublin Ireland) according to the manufacturer's instructions. Labelled samples were combined and co-hybridised on equine cDNA microarrays using SlideHyb Glass Array Hybridisation Buffer #3 (Applied Biosystems, Cambridgeshire, UK). Microarray hybridisations were performed on an automated HS400 hybridisation station (Tecan Group Ltd. Seestrasse 103 CH-8708 Männedorf, Switzerland) with the following protocol - wash: 75°C, runs 1, wash 10 s, soak 20 s; probe injection: 85°C; denaturation: 95°C, 2 min; hybridisation: 42°C, agitation frequency medium, 4 h; hybridisation: 35°C, agitation frequency medium, 4 h; hybridisation: 30°C, agitation frequency medium, 4 h; wash: 37°C, runs 2, wash 10 s, soak 20 s; wash: 25°C, runs 2, wash 15 s, soak 30 s; wash: 25°C, runs 2, wash 20 s, soak 40 s; slide drying: 25°C 2 min.

The experimental design was a direct comparison for each animal between pre- and both post-exercise time points. Each slide was hybridised with samples from T_0 _*Vs *T_1 _and from T_0 _*Vs *T_2 _for each animal. Technical replicates in the form of a dye swap were performed for each investigation.

### Microarray scanning and data acquisition

Hybridised and dried slides were scanned using a GenePix 4000B scanner (Molecular Devices, Berkshire, UK) and image acquisition, first-pass data analysis and filtering were carried out using the GenePix 6.0 microarray image analysis package (Molecular Devices, Berkshire, UK). As a first step of feature extraction spots that were flagged as 'poor' by the GenePix software (due to signal foreground or background contamination, shape irregularity or poor spot quality) were assigned a weight of zero and were excluded from differential expression analyses. Images of the slides were visually examined and any obvious irregularities were also flagged, assigned a weight of zero and excluded from differential expression analyses. All spots with a mean signal of less than background plus two standard deviations were flagged and were also excluded from differential expression analyses.

### Microarray data analyses

All statistical analyses on the gene expression data were performed using the R language, version 2.5.1 [105] and the packages statmod and LIMMA from the Bioconductor project [106]. Robust multichip average (RMA) [107] and print tip lowess normalization [108,109] were performed on the data before differential expression analyses were performed using the lmFit function in LIMMA. Fluor647:Fluor555 log_2 _ratios were calculated for each spot on the microarray and duplicate spots were averaged. The function duplicateCorrelation [110] was used to estimate the correlation between technical replicates (dye swaps) by fitting a mixed linear model by REML individually for each probe. The function also returned a consensus correlation, which is a robust average of the individual correlations. This was used to fit a linear model to the expression data for each probe taking into account the inter-technical replicate correlation between each microarray hybridisation.

Differentially expressed targets were determined using a Bayes moderated t-test [111]. Multiple testing was addressed by controlling the false discovery rate (FDR) using the correction of Benjamini and Hochberg [112]. A probe was flagged as differentially expressed if the corrected *P *value was < 0.05.

### Functional clustering according to gene ontology annotations

A list of Entrez IDs of human homologs of probes on the microarray was obtained in a similar manner as for microarray annotation. Using the Entrez IDs of human homologues of equine genes it was possible to use the Database for Annotation, Visualization and Integrated Discovery (DAVID) [28,29] for functional clustering and overrepresentation analyses. DAVID was used to investigate the representation of broad gene ontology (GO) categories (Level 1) on the equine cDNA microarray relative to the whole genome.

The DAVID system was also used to cluster differentially expressed genes according to their function. For T_0 _*vs *T_2 _experiments, a probe was called differentially expressed if its corrected *P *value was < 0.05 [112]. The enrichment of categories was assessed and compared with the proportion observed in the total population of genes on the microarray, using the Expression Analysis Systematic Explorer (EASE) tool within DAVID [113]. A different approach was used when functionally clustering differentially expressed genes from the T_0 _*vs *T_1 _experiments. Although we expected there would only be small number of genes differentially expressed at T_1_, there remains the possibility that some more modest but still genuine changes in gene expression may not be detected. Therefore the FatiScan [56,114] gene enrichment test was used to analyse the transcriptional profile immediately after exercise. FatiScan is part of the Babelomics Suite Genes and tests for the asymmetrical distribution of biological labels in an ordered list of genes. Genes were ranked by differential expression and FatiScan was used to detect functional blocks (GO and KEGG pathways) that were significantly up-regulated and down-regulated immediately after exercise.

### Quantitative real time RT-PCR

Selected cDNA samples from seven of the eight animals were quantified by real time qRT-PCR. One of the animals was omitted due to a shortage of RNA in the pre-exercise sample. 1 μg of total RNA from each sample was reverse transcribed into cDNA with oligo-dT primers using a SuperScript™ III first strand synthesis SuperMix kit according to the manufacturer's instructions (Invitrogen Ltd, Paisley, UK). The converted cDNA was diluted to 2.5 ng/μl working stocks and stored at -20°C for subsequent analyses. Oligonucleotide primers for real time qRT-PCR were designed using Primer3 version 3.0 http://www.primer3.sourceforge.net and commercially synthesized (MWG Biotech, Germany), details of these primers are available in additional file 5. Each reaction was carried out in a total volume of 20 μl with 2 μl of cDNA (2.5 ng/μl), 10 μl SYBR^® ^Green PCR Master Mix (Applied Biosystems, Cambridgeshire, UK) and 8 μl primer/H_2_O. Optimal primer concentrations were determined by titrating 50, 300 and 900 nM final concentrations and disassociation curves were examined for the presence of a single product. qRT-PCR was performed using a 7500 Fast Real-Time PCR machine (Applied Biosystems, Cambridgeshire, UK).

A panel of four putative reference or 'housekeeping' genes were selected for a reference gene study. This panel comprised two frequently used reference genes (*HPRT1*, hypoxanthine phosphoribosyltransferase 1 gene; and *GAPDH*, glyceraldehyde-3-phosphate dehydrogenase gene) and two genes (*NSUN6*, NOL1/NOP2/Sun domain family, member 6 gene; and *PIGO*, phosphatidylinositol glycan anchor biosynthesis, class O gene) that were selected based on minimal variation across the time points observed in the microarray results. The panel of genes was evaluated using geNorm version 3.4 for Microsoft Excel [115]. Briefly, the gene expression stability measure *'M' *for each control gene was calculated as the pairwise variation for that gene with all other tested reference genes across the exercise time-course (T_0_, T_1_, T_2_). The candidate reference genes were ranked in order of decreasing '*M' *values or increasing mRNA expression stability [85]. Based on the geNorm analyses, the *NSUN6 *gene was the optimal reference gene and pre-exercise (T_0_) values were used to normalise the data. The 2^-ΔΔCT ^method (where CT is cycle threshold) was used to determine mean fold changes in gene expression [116]. The Student's t-test was used to identify significant differences in mRNA abundance between time-points.

## Authors' contributions

EH, DMacH and LK conceived and designed the experiments. LK coordinated and performed the exercise experiment. BMcG, EH, LK and SE participated in the exercise experiment and collection of samples. SE performed the RNA extractions. JMacL provided the microarrays. BMcG and GOG performed the microarray and qRT-PCR experiments. SP was responsible for the annotation of the microarray. BMcG and SP analysed the data. DMacH assisted with manuscript preparation. BMcG and EH wrote the paper. All authors read and approved the final manuscript.

## Supplementary Material

Additional file 1 **The relative distributions of gene ontology (GO) categories (Level 1) on the equine cDNA microarray**. A list of all available human orthologues to equine genes was compared to a list of human orthologues of probes on the microarray using the Database for Annotation, Visualization and Integrated Discovery (DAVID) [28,29] for functional clustering and overrepresentation analyses. The gene ontologies represented on the graph are: 1) gene expression, 2) metabolic process, 3) cellular process, 4) membrane-enclosed lumen, 5) macromolecular complex, 6) organelle part, 7) organelle, 8) cell part, 9) catalytic activity, 10) binding. Click here for file

Additional file 2 **Gene expression a T**_1_. Excel file containing GenBank IDs, log fold changes, unadjusted and adjusted p-values and annotation where available for all probes on the array. Click here for file

Additional file 3 **Gene expression a T**_2_. Excel file containing GenBank IDs, log fold changes, unadjusted and adjusted p-values and annotation where available for all probes on the array. Click here for file

Additional file 4 **Genes differentially expressed in KEGG pathways over-represented at T_2_**. Click here for file

Additional file 5 **Equine oligonucleotide primers used for real time qRT-PCR**. Click here for file
