# Supplementary figures and images for: Transcriptional adaptations following exercise in Thoroughbred horse skeletal muscle highlights molecular mechanisms that lead to muscle hypertrophy
Source: BMC Genomics. 2009 Dec 30;10:638. doi: 10.1186/1471-2164-10-638 (PMC2812474; doi:10.1186/1471-2164-10-638)

## Slide 1
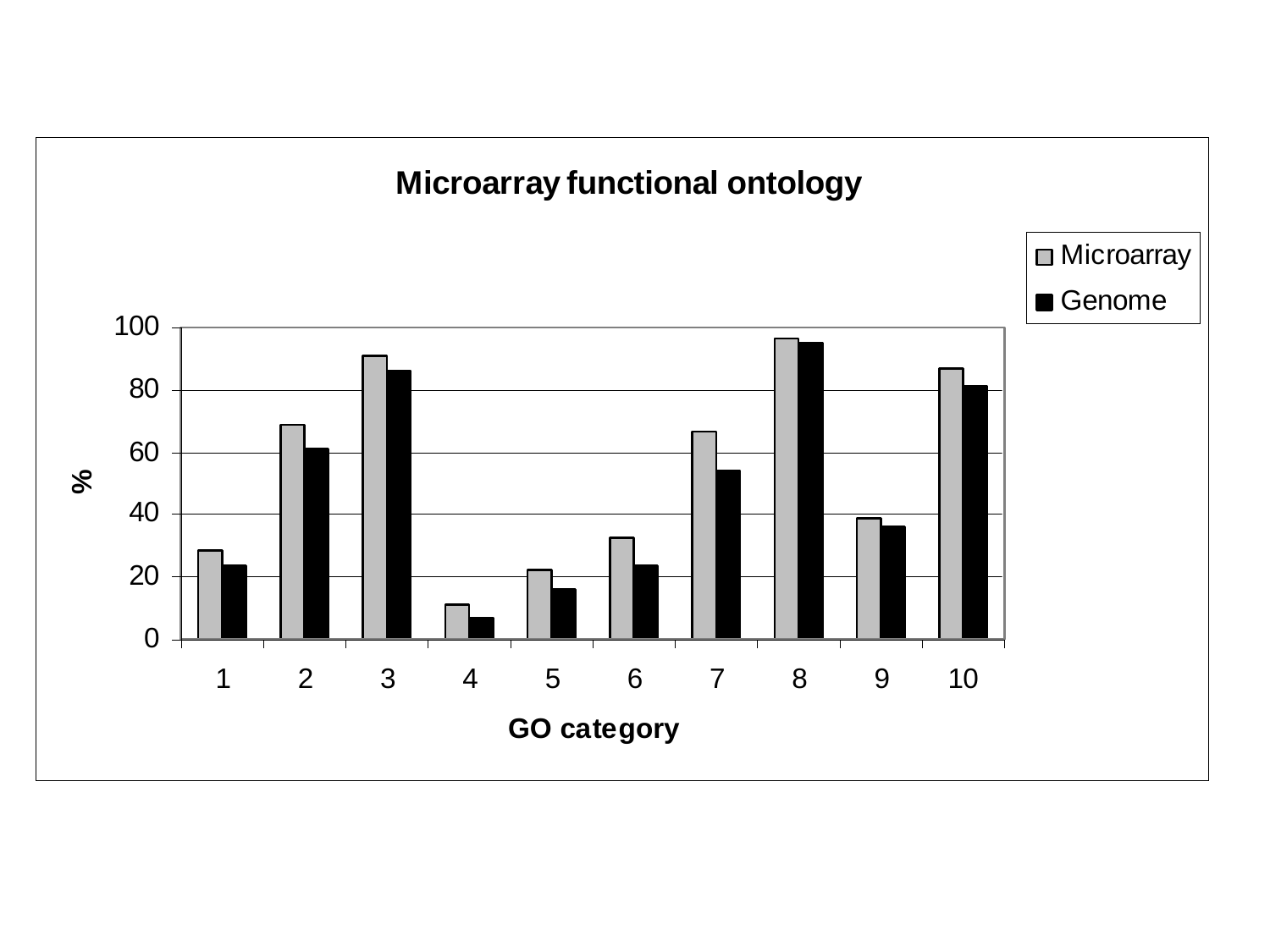

Supplement: Additional file 1 — The relative distributions of gene ontology (GO) categories (Level 1) on the equine cDNA microarray. A list of all available human orthologues to equine genes was compared to a list of human orthologues of probes on the microarray using the Database for Annotation, Visualization and Integrated Discovery (DAVID) [28,29] for functional clustering and overrepresentation analyses. The gene ontologies represented on the graph are: 1) gene expression, 2) metabolic process, 3) cellular process, 4) membrane-enclosed lumen, 5) macromolecular complex, 6) organelle part, 7) organelle, 8) cell part, 9) catalytic activity, 10) binding. [file 1471-2164-10-638-S1.PPT]
